# Activities of different types of Thai honey on pathogenic bacteria causing skin diseases, tyrosinase enzyme and generating free radicals

**DOI:** 10.1186/0717-6287-48-4

**Published:** 2015-01-16

**Authors:** Kanyaluck Jantakee, Yingmanee Tragoolpua

**Affiliations:** Department of Biology, Faculty of Science, Chiang Mai University, Chiang Mai, 50200 Thailand

**Keywords:** Anti-bacterial activity, Pathogenic bacteria, Anti-free radicals, Anti-tyrosinase

## Abstract

**Background:**

Honey is a natural product obtained from the nectar that is collected from flowers by bees. It has several properties, including those of being food and supplementary diet, and it can be used in cosmetic products. Honey imparts pharmaceutical properties since it has antibacterial and antioxidant activities. The antibacterial and antioxidant activities of Thai honey were investigated in this study.

**Results:**

The honey from longan flower (source No. 1) gave the highest activity on MRSA when compared to the other types of honey, with a minimum inhibitory concentration of 12.5% (v/v) and minimum bactericidal concentration of 25% (v/v).

Moreover, it was found that MRSA isolate 49 and *S. aureus* were completely inhibited by the 50% (v/v) longan honey (source No. 1) at 8 and 20 hours of treatment, respectively. Furthermore, it was observed that the honey from coffee pollen (source No. 4) showed the highest phenolic and flavonoid compounds by 734.76 mg gallic/kg of honey and 178.31 mg quercetin/kg of honey, respectively. The antioxidant activity of the honey obtained from coffee pollen was also found to be the highest, when investigated using FRAP and DPPH assay, with 1781.77 mg FeSO_4_•7H_2_O/kg of honey and 86.20 mg gallic/kg of honey, respectively. Additionally, inhibition of tyrosinase enzyme was found that honey from coffee flower showed highest inhibition by 63.46%.

**Conclusions:**

Honey demonstrates tremendous potential as a useful source that provides anti-free radicals, anti-tyrosinase and anti-bacterial activity against pathogenic bacteria causing skin diseases.

## Background

Infection of skin by pathogenic bacteria is one of the major health concerns in Thailand as well as the rest of the world. Skin and wound infections are frequently caused by pathogenic bacteria such as *Staphylococcus aureus*, *Staphylococcus epidermidis*, *Micrococcus luteus*, *Streptococcus pyogenes*, *Pseudomonas aeruginosa*, *Escherichia coli*, *Klebsiella pneumoniae,* methicillin resistant or sensitive *S. aureus* (MRSA or MSSA), and vancomycin resistant *Enterococci*[1–3]. The development of resistant bacterial strains resulting from excessive use of antibiotics has limited the efficacy of currently available antibiotics. Therefore, it is imperative that new pharmaceutical agents are developed for the effective treatment of diseases.

Honey is a natural product obtained from the nectar that is collected from flowers by bees. It has several properties, including those of being food and supplementary diet, and it can be used in cosmetic products. Honey imparts pharmaceutical properties since it has antibacterial and antioxidant activities. Therefore, honey has been in use since ancient times as medicine in a wide variety of treatments [2]. In addition to high sugar and low water activity content (a_w_), honey also contains bioactive compounds and various enzymes [4]. Honey has been reported to exhibit gastroprotective, antibacterial, antifungal, antioxidant, and anti-inflammatory properties [5–8]. It has been demonstrated to be effective against several human bacterial pathogens, including *Escherichia coli*, *Enterobacter aerogenes*, *Salmonella typhimurium,* and *Staphylococcus aureus*[9]. The antibacterial activities of honey were demonstrated by using osmotic effects, acidity, hydrogen peroxide generation, and phytochemical components [3]. The hydrogen peroxide, which is produced by the enzyme glucose oxidase, a major antibacterial agent, showed different concentrations in the different types of honey. In addition, it was found that some honey consisted of many natural antibacterial components. The well-studied New Zealand mānuka honey contained 1,2 dicarbonyl compound methylglyoxal (MGO), or the unique mānuka factor (UMF), and the cationic antimicrobial peptide bee defensin-1, which were identified as specific antibacterial substances [10, 11]. Several publications have shown the antimicrobial properties of mānuka honey and its activity against pathogenic bacteria including *Staphylococcus aureus*, *Pseudomonas aeruginosa,* and *Escherichia coli*[12, 13]. In addition, honey contains many substances including phenolic acids, flavonoids, ascorbic acid, protein, carotenoid, and enzymes [14, 15]. Phenolic and flavonoids compounds are some of the most important groups of the antioxidant substances present in honey. The diversity of the phenolic content and the antioxidant capacity depends on the processing, handling, and storage of honey [16]. Moreover, many phytochemicals in natural substances have been shown as skin-whitening agents by inhibition of tyrosinase enzyme in melanin synthesis pathway [17]. In the biosynthetic pathway of melanin formation, tyrosinase has a primary role in the reaction by oxidation of tyrosine to L-3,4-dihydroxyphenylalanine (L-DOPA) and dopaquinone [18].

In Thailand, various types of honey are produced and consumed on a large scale; however, only a few pieces of research literature and information on the biological properties of honey have been reported. For that reason, this study aimed to investigate the efficacy of different types of Thai honey on inhibition of pathogenic bacteria on skin. Additionally, the polyphenol, flavonoid, antioxidant and anti-tyrosinase properties of honey were determined.

## Results

### Viscosity and pH of honey

The viscosity and pH values were shown in Table 1. The pH value of honeys ranged from 3.37 ± 0.08 to 4.06 ± 0.03. Additionally, honeys showed the viscosity of 1,083.50 ± 12.02 to 4,892.00 ± 63.64 Centipoise. Mānuka honey showed very high viscosity so the viscosity could not be measured by the viscometer used in this study.

**Table 1:** **pH and viscosity values of honey**

Source	Type of pollen	Vicosity (cP)	pH
**1**	**Longan**	2599.50 ± 3.54	4.02 ± 0.06
	**Lychee**	1597.50 ± 19.09	4.06 ± 0.03
	**Polyflora**	2940.00 ± 25.46	3.87 ± 0.11
**2**	**Longan**	3570.50 ± 47.38	3.98 ± 0.02
	**Sunflower**	2624.00 ± 9.90	3.77 ± 0.12
	**Polyflora**	2824.00 ± 41.01	3.98 ± 0.07
**3**	**Longan**	3481.50 ± 58.69	3.87 ± 0.10
	**Lychee**	3332.50 ± 106.77	4.02 ± 0.04
	**Polyflora**	2993.00 ± 42.43	3.84 ± 0.05
	**Forest flora**	1595.00 ± 28.28	3.88 ± 0.12
**4**	**Coffee**	1083.50 ± 12.02	4.00 ± 0.09
**5**	**Polyflora**	1641.00 ± 14.14	3.84 ± 0.07
**6**	**Lychee**	1759.50 ± 21.92	3.37 ± 0.08
	**Sunflower**	2669.50 ± 27.58	3.76 ± 0.08
	**Polyflora**	4892.00 ± 63.64	3.79 ± 0.16
	**Sesame**	2953.00 ± 53.74	3.57 ± 0.04
**Mānuka honey**		-	3.85 ± 0.06

### Determination of activity of honey on bacteria using the agar well diffusion assay

The results of the antibacterial assays of 16 types of honey from 6 sources are shown in Table 2. The honey samples at the concentration of 100% inhibited *S. aureus* and MRSA*.* However, *Corynebacterium* sp. was not inhibited by honey obtained from the pollens of forest flora (source No.3), and honey obtained from the pollens of lychee, sunflower, and sesame (source No.6). In addition, honey from the pollens of longan, lychee, and polyflora (source No.1) and honey from polyflora (source No.5, No.6) showed activity on *P. acnes*. Moreover, all the tested bacteria were found to be inhibited by mānuka honey. Upon testing the inhibiton of bacteria with honey obtained from the longan pollen (source No.3), it was found that the antibacterial activity against MRSA 49 was higher than that exhibited by mānuka honey, with diameters of the inhibition zone of 24.00 ± 5.20 mm. However, after treatment of all Thai honey that used in this study with catalase enzyme, the inhibition zones were not observed except the treatment of mānuka honey. *S. aureus, Corynebacterium* sp., MRSA 49, MRSA 50 and *P. acne*s were inhibited by mānuka honey after treatment with catalase enzyme with diameters of the inhibiton zones of 20.33 ± 0.58, 24.00 ± 1.00, 23.33 ± 2.08, 20.33 ± 0.58 and 11.33 ± 0.58 mm., respectively.

**Table 2:** **Effect of honey on growth of pathogenic bacteria causing skin disease by agar well diffusion method**

Source	Type of pollen	Zone of Inhibition (mm)				
		Bacterial strain				
		***S. aureus***	MRSA 49	MRSA 50	***Corynebacterium*** sp.	***P.acnes***
**1**	**Longan**	15.67 ± 1.15	19.67 ± 1.53	21.33 ± 2.31	11.66 ± 0.58	8.67 ± 0.58
	**Lychee**	12.00 ± 0.00	11.33 ± 1.53	13.33 ± 2.52	6.50 ± 0.00	9.33 ± 0.58
	**Polyflora**	14.33 ± 1.15	17.33 ± 1.53	16.33 ± 1.15	9.33 ± 1.15	9.67 ± 0.58
**2**	**Longan**	13.00 ± 1.00	16.33 ± 0.58	15.00 ± 0.00	11.00 ± 0.00	0.00 ± 0.00
	**Sunflower**	9.67 ± 0.58	11.33 ± 0.53	10.33 ± 0.58	7.00 ± 0.00	0.00 ± 0.00
	**Polyflora**	11.67 ± 0.58	15.33 ± 0.58	15.00 ± 1.00	10.00 ± 0.00	0.00 ± 0.00
**3**	**Longan**	18.33 ± 0.58	24.00 ± 5.20	22.67 ± 0.58	16.00 ± 1.00	0.00 ± 0.00
	**Lychee**	13.330.58	19.33 ± 1.15	17.33 ± 2.52	10.33 ± 0.58	0.00 ± 0.00
	**Polyflora**	14.33 ± 1.15	20.33 ± 2.33	19.67 ± 1.53	9.33 ± 0.58	0.00 ± 0.00
	**Forest flora**	0.00 ± 0.00	0.00 ± 0.00	0.00 ± 0.00	0.00 ± 0.00	0.00 ± 0.00
**4**	**Coffee**	13.00 ± 1.00	16.00 ± 0.00	15.33 ± 0.58	11.00 ± 1.00	0.00 ± 0.00
**5**	**Polyflora**	13.67 ± 1.15	21.00 ± 3.61	13.67 ± 1.15	11.67 ± 0.58	8.33 ± 0.58
**6**	**Lychee**	10.00 ± 0.00	10.67 ± 0.58	11.33 ± 0.58	0.00 ± 0.00	0.00 ± 0.00
	**Sunflower**	0.00 ± 0.00	0.00 ± 0.00	0.00 ± 0.00	0.00 ± 0.00	0.00 ± 0.00
	**Polyflora**	13.67 ± 1.15	14.00 ± 1.00	17.33 ± 1.15	10.33 ± 0.58	7.33 ± 0.58
	**Sesame**	0.00 ± 0.00	10.33 ± 0.58	10.00 ± 0.00	0.00 ± 0.00	0.00 ± 0.00
**Mānuka honey**		20.33 ± 0.58	19.67 ± 2.52	22.00 ± 1.00	22.00 ± 3.00	11.67 ± 0.58
**Control solution**		0.00 ± 0.00	0.00 ± 0.00	0.00 ± 0.00	0.00 ± 0.00	0.00 ± 0.00
**Gentamycin 2 mg/ml**		23.00 ± 0.00	0.00 ± 0.00	0.00 ± 0.00	28.33 ± 0.58	34.00 ± 1.00
**Gentamycin 10 mg/ml**		ND	9.67 ± 0.58	10.00 ± 0.00	ND	ND

Note: The data are given as mean ± standard deviation (SD) of triplicate experiments. The statistical comparison between values from the different types of honey and the bacterial strains was done using post hoc Duncan’s test. The values were found to be significantly different (p < 0.05) when compared between different strains of bacteria and different types of honey. ND = not determined.

Phenol was also used as positive control. After treatment *S. aureus, Corynebacterium* sp., MRSA 49, MRSA 50 and *P. acne*s by phenol at 12%, the inhibition zones were 25.67 ± 0.58, 25.33 ± 1.52, 21.00 ± 1.73, 23.00 ± 3.60 and 28.67 ± 0.58 mm., respectively. In addition, it was observed that *M. luteus*, *B. subtilis*, *S. epidermidis,* and *Ps. aeruginosa* were not inhibited by honey.

### Determination of minimum inhibitory concentration (MIC) and minimum bactericidal concentration (MBC) of honey on bacteria

The MIC and MBC values of honey obtained from the different pollens and the MIC and MBC values of mānuka honey were determined and compared for antibacterial activity using the broth dilution method. Most honey showed antibacterial activity against all of the pathogenic bacteria. The MIC values of honey ranged from 12.5% to 50%, and the MBC values ranged from 25% to >50%,while the MIC values observed from mānuka honey were lower, and ranged between 3.125% and 25% (Table 3). However, the honey obtained from the longan and polyflora pollens (source No.1) showed lower MIC and MBC values of 12.5% and 25%, respectively, on MRSA 49 and MRSA 50 when compared to the other varieties of honey.

**Table 3:** **Minimum inhibitory concentration (MIC) and minimum bactericidal concentration (MBC) values of honey against pathogenic bacteria causing skin disease**

Source	Type of pollen	MIC and MBC (% honey)									
		Bacterial strains									
		***S. aureus***		MRSA 49		MRSA 50		***Corynebacterium sp.***		***P.acnes***	
		MIC	MBC	MIC	MBC	MIC	MBC	MIC	MBC	MIC	MBC
**1**	**Longan**	25	25	12.5	25	12.5	25	25	25	25	50
	**Lychee**	25	25	25	50	25	25	25	50	25	50
	**Polyflora**	25	25	25	25	25	25	12.5	25	25	50
**2**	**Longan**	25	50	25	25	25	25	25	50	25	50
	**Sunflower**	25	50	25	50	25	25	25	50	25	50
	**Polyflora**	25	>50	25	25	25	50	25	50	25	50
**3**	**Longan**	25	50	25	25	25	50	25	50	25	50
	**Lychee**	25	50	25	25	25	50	25	50	25	50
	**Polyflora**	25	50	25	25	25	25	25	50	25	50
	**Forest flower**	25	>50	25	>50	25	>50	25	>50	25	>50
**4**	**Coffee**	25	25	25	25	25	50	12.5	50	25	50
**5**	**Polyflora**	25	50	25	25	25	25	12.5	50	50	50
**6**	**Lychee**	25	>50	25	>50	25	50	25	50	25	50
	**Sunflower**	25	>50	25	>50	25	>50	25	>50	50	>50
	**Polyflora**	25	50	25	50	25	50	25	50	25	50
	**Sesame**	25	50	25	>50	25	50	25	50	50	50
**Mānuka honey**		6.25	6.25	6.25	6.25	6.25	6.25	3.125	6.25	25	25

### Determination of time–kill endpoints of honey on bacterial growth

**Figure 1:**
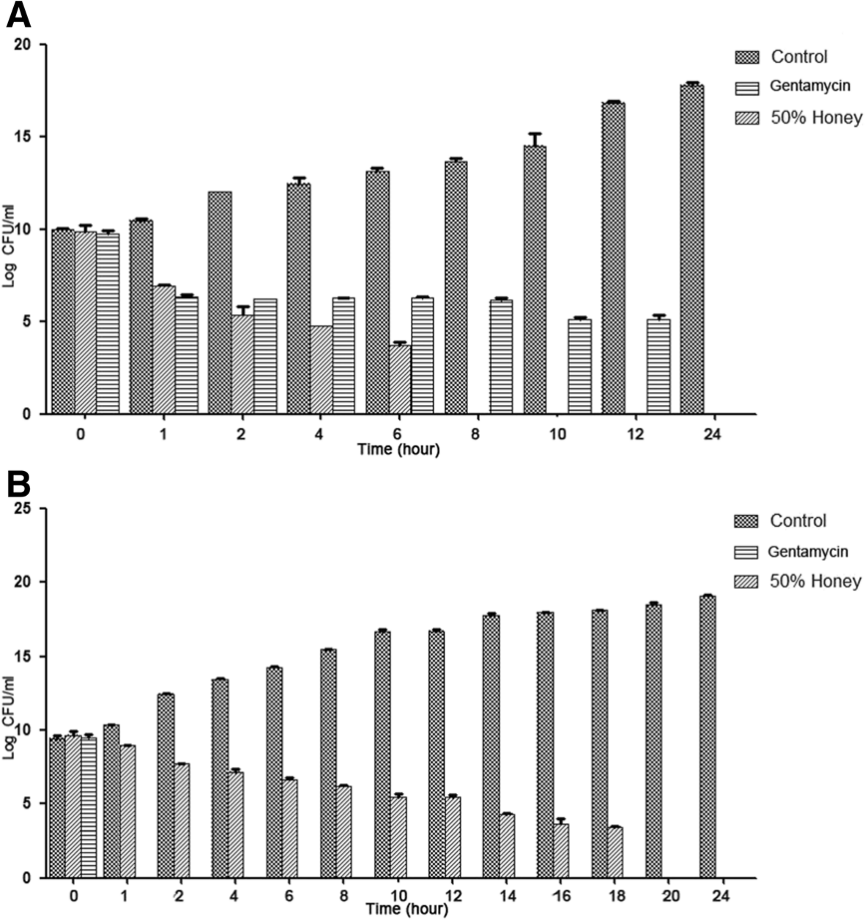
**The time–kill kinetic of the 50% honey from longan flowers (source No. 1) on MRSA49 (A) and** ***S. aureus*** **(B).**

The time–kill assay of honey obtained from longan flowers (source No.1) at a concentration of 50% (v/v) was performed against *S.aureus* and MRSA49 after treatment for 10 minutes, 20 minutes, 30 minutes, 60 minutes, 90 minutes, and 120 minutes, and every 2 hours until 24 hours. The results of the bacterial growth for each time are presented in Figure 1. The MRSA49 growth was completely inhibited by the 50% honey at 8 hours, while the *S.aureus* growth was completely inhibited at 20 hours after the treatment.

### Determination of total phenolic content of honey

The total phenolic compounds were determined using the Folin–Ciocalteu method and reported as gallic acid equivalents with reference to the standard curve (y = 8.8325x- 0.0433, R^2^ = 0.9981). The honey from coffee flower (source No.4) had, significantly, the highest total phenolic content (p < 0.05), of 734.76 mg gallic/kg of honey, when compared to the other types of honey, except mānuka honey (Table 4).

**Table 4:** **The phenolic and flavonoid contents and antioxidant activities of honey by DPPH and FRAP assays**

Source	Type of pollen	Phenolic content (mg GAE/kg of honey)	Flavonoid content (mg quercetin /kg of honey)	DPPH (mg GAE/kg of honey)	FRAP (mg FeSO _4_ /kg of honey)
**1**	**Longan**	301.93 ± 47.90	40.46 ± 11.49	14.04 ± 0.18	627.57 ± 91.61
	**Lychee**	253.69 ± 50.34	59.77 ± 20.93	7.35 ± 0.10	440.16 ± 57.85
	**Polyflora**	320.37 ± 30.78	66.56 ± 15.64	12.28 ± 0.28	295.60 ± 50.86
**2**	**Longan**	467.66 ± 16.19	81.91 ± 3.09	33.74 ± 2.90	899.37 ± 119.27
	**Sunflower**	380.94 ± 14.37	67.23 ± 4.70	17.93 ± 0.44	860.40 ± 124.53
	**Polyflora**	425.19 ± 58.77	51.62 ± 1.41	15.06 ± 0.29	568.12 ± 95.83
**3**	**Longan**	312.53 ± 32.54	53.88 ± 2.62	11.36 ± 0.25	696.83 ± 94.80
	**Lychee**	280.44 ± 64.79	34.48 ± 1.17	5.93 ± 0.02	319.56 ± 28.41
	**Polyflora**	350.05 ± 53.06	55.42 ± 2.23	17.03 ± 2.54	645.61 ± 87.77
	**Forest flower**	315.72 ± 12.37	35.40 ± 1.46	7.10 ± 0.12	373.76 ± 52.45
**4**	**Coffee**	734.76 ± 155.50	178.31 ± 40.04	86.20 ± 0.64	1781.78 ± 218.70
**5**	**Polyflora**	674.81 ± 52.77	79.75 ± 1.70	48.18 ± 9.20	1016.11 ± 87.91
**6**	**Lychee**	311.87 ± 12.11	55.99 ± 2.17	8.71 ± 1.86	269.83 ± 9.20
	**Sunflower**	274.89 ± 69.37	47.36 ± 2.84	13.39 ± 2.86	490.61 ± 44.47
	**Polyflora**	234.66 ± 22.47	29.86 ± 6.69	6.15 ± 0.65	331.87 ± 17.50
	**Sesame**	314.92 ± 16.82	54.97 ± 0.19	9.12 ± 1.05	279.76 ± 19.65
**Mānuka honey**		831.88 ± 120.26	162.87 ± 11.51	83.80 ± 11.29	954.10 ± 123.77

Note: The data are given as mean ± standard deviation (SD) of triplicate experiments. The statistical comparison between values from the different types of honey applied using the post hoc Duncan test. The values were found to be significantly different (p < 0.05).

### Determination of flavonoid content of honey

The total flavonoid content of the honey samples were in the range of 29.86 mg quercetin/kg of honey to 178.31 mg quercetin/kg of honey, which was expressed using quercetin as the standard (Table 4). The honey from coffee flower (source No.4) showed significantly high flavonoid content, of 178.31 mg quercetin/kg of honey. Therefore, it can be concluded that the level of the flavonoid content in a honey sample is dependent on the type of flora.

### Determination of antioxidant activity of honey by 2, 2-diphenyl-1-picrylhydrazyl (DPPH) assay

The DPPH radical scavenging analysis was used to investigate the overall hydrogen or electron donating activity of single antioxidants. The scavenging abilities of the different varieties of honey are presented in Table 4. The results demonstrated that the greatest antioxidant activity of 86.20 mg gallic/kg of honey was found in the honey collected from the nectar of coffee flower (source No.4), while the honey obtained from the lychee flower (source No.3) demonstrated the lowest DPPH radical scavenging activity (5.93 mg gallic/kg of honey).

### Determination of antioxidant activity of honey by ferric reducing antioxidant power assay (FRAP)

The FRAP assay was used to estimate the amount of antioxidants or reductants in a sample. The assay is based on the ability of the sample to reduce the Fe^3+^ to the Fe^2+^ couple, which was applied to determine the differences in the antioxidant profiles of the various types of honey. The results of the FRAP assay and the values are presented in Table 4. The honey from coffee flower (source No.4) had, significantly, the highest value of antioxidant ability, with 1781.78 mg FeSO_4_/kg of honey.

### Determination of anti-tyrosinase activity of honey

The tyrosinase inhibitory activity of honeys is shown in Table 5. The inhibitory effects on tyrosinase activity ranged from 22.71 ± 6.82 - 87.73 ± 5.33%. The highest activity of tyrosinase inhibition was found from mãnuka honey. The tyrosinase inhibition activity of mãnuka honey was expressed as 500.15 mg koji/kg honey (p < 0.05). However, honey from coffee flower (source No. 4) had highest anti-tyrosinase activity, when compared to the other types of tested honey (p < 0.05).

**Table 5:** **Anti-tyrosinase activity of honey from different types of pollen**

Source	Type of pollen	Tyrosinase inhibition (%)	Kojic acid equivalents (mg koji/kg honey)
**1**	**Longan**	60.09 ± 5.51	34.23
	**Lychee**	56.51 ± 1.44	24.19
	**Polyflora**	58.20 ± 4.76	28.50
**2**	**Longan**	53.27 ± 10.38	17.67
	**Sunflower**	46.64 ± 3.85	9.29
	**Polyflora**	45.01 ± 2.78	7.93
**3**	**Longan**	45.02 ± 8.01	7.94
	**Lychee**	48.30 ± 9.65	10.91
	**Polyflora**	40.17 ± 4.44	4.96
	**Forest flower**	22.71 ± 6.82	0.91
**4**	**Coffee**	63.46 ± 16.30	47.49
**5**	**Polyflora**	44.57 ± 4.70	7.60
**6**	**Lychee**	51.87 ± 5.94	15.42
	**Sunflower**	52.12 ± 4.05	15.81
	**Polyflora**	49.61 ± 11.26	12.40
	**Sesame**	52.53 ± 5.19	16.45
**Mānuka honey**		87.73 ± 5.33	500.15

Note: The data are given as mean ± standard deviation (SD) of triplicate experiments. The statistical comparison between values from different types of honey applied using the post hoc Duncan test. The values were found to be significantly different (p < 0.05).

## Discussion

In this study, Thai honey from different types of floral and sources were collected. The physical characteristics of honey were observed by determination of pH and viscosity in this study. All types of honey showed pH ranging from 3.37 ± 0.08 to 4.06 ± 0.03 which were similar to the previous reports that various types of honey also showed acidic pH. Moreover, honey showed the viscosity that ranged from 1,083.50 ± 12.02 to 4,892.00 ± 63.64 Centipoise. The viscosity of honey was influenced by temperature, moisture content and the presence of crystals and colloids. Honey from different sources of flora showed the different physical characteristics such as liquid honey or creamed honey because the honey contained wide ranges of compounds [19–21].

In this research, we found that all honey had antibacterial activity against *S. aureus*, MRSA, and *Corynebacterium* sp. and *P. acnes* by the agar well diffusion assay. However, all the varieties of honey were observed to exhibit the greatest inhibition on the growth of MRSA. The high viscosity of honey helped to provide the barrier and protect bacterial infection in the host [22]. Moreover, the high sugar content of the honey could affect the osmolarity and inhibited microbial growth [4].

Additionally, the antibacterial activity of mānuka honey (*Leptospermum scoparium*) against *S. aureus*, MRSA, and *Pseudomonas* sp. were demonstrated. The antibacterial activity of honey depended on various factors that function either singularly or synergistically [15, 23, 24]. The honey consists of hydrogen peroxide, phenolic compounds, lower pH, osmotic pressure, and other phytochemical content. Honey has the ability to generate hydrogen peroxide-related antimicrobial activity. The production of hydrogen peroxide by transforming glucose substrate with the glucose oxidase of honey depends on the enzyme level and the floral sources of honey [19].

The result also revealed that the antibacterial activity was associated with the region where the honey was produced, as well. Different varities of honey from different countries and regions vary widely and significantly in their antibacterial activity and in their action against pathogenic bacteria. Most of gram positive bacteria; *S. aureus*, MRSA, *Corynebacterium* sp., and *P. acnes* were inhibited by the honey from different types of pollen. Therefore, the action of honey on tested organism may be due to the difference in the species of bacteria. In this study, the mode of action of Thai honey against the bacteria depended on different factors such as the osmolarity, acidity and hydrogen peroxide generation. The formation of free free OH^-^ radicals can break DNA and oxidize thiol-groups of proteins and lipids leaded to damage of bacterial cells. Moreover, after treatment of Thai honey that used in this study with catalase enzyme resulted in the removal of the antibacterial activity whereas the antibacterial activity of mānuka honey still retained.

In addition, the broth dilution method was used to determine the MIC and MBC values because this method had more efficiency to indicate quantitative results compared to the agar well on diffusion method [5, 24]. Minimum inhibitory concentration (MIC) and minimum bactericidal concentration (MBC) of honey determine the antibacterial activity of honey. The MIC values of all the varieties of honey (Table 3) were found to be in the range of 12.5–50% and the MBC values were found to be in the range of 25% to >50% (Table 3). Mānuka honey showed higher antibacterial activity, with MIC ranging from 3.125% to 25%. In this study, the broth dilution method, bacteria were brought into direct contact with honey, while in the agar well diffusion method, the components relied on diffusion through the agar. Moreover, the diluted honey in the broth dilution method could generate hydrogen peroxide since the enzyme glucose oxidase oxidized glucose to gluconic acid and hydrogen peroxide. Therefore, antibacterial activity of the different varieties of honey was from hydrogen peroxide activity [25].

However, other studies showed that the inhibitory activity of honey on pathogenic bacteria was observed when the treatment of the bacteria with honey was conducted at concentrations lower than 3% or at concentration 50% or higher [24, 26]. Furthermore, mānuka honey, as previous reports have pointed out, demonstrates high antimicrobial activity and is known to contain non-peroxide components such as polyphenols and protein defensin-1 that exhibit antimicrobial activity [27]. Additionally, methylglyoxal was also found as a major compound in mānuka honey and could inhibit pathogenic bacteria such as *E. coli* and *S. aureus*[11]. Besides, honey from the flowers of *E. cladocalyx* (Blue gum), *Erica species* (Fynbos), and *L. cordifolium* (Pincushion) were seen to demonstrate antibacterial activity similar to mānuka honey [28].

The investigation of the killing kinetic of honey obtained from the longan flower at the concentration of 50% (v/v) demonstrated the reduction in the survival of *S. aureus* and MRSA49, although *S. aureus* exhibited more resistance to honey. Interestingly, methicillin resistant *S. aureus* (MRSA) was found to have been completely inhibited by the honey at 8 hours of treatment. Therefore, the significant finding of the study was the potent activity of Thai honey against antibiotic-resistant pathogenic bacteria, MRSA. The anti-bacterial activity of the honey is a property that is of valuable interest in its function as an agent in treating bacterial infections that are resistant to the action of antibiotics.

Another study conducted using Revamil^®^ medical-grade honey (RS honey) produced in the Netherlands at a concentration of 40% (v/v) also demonstrated anti-bacterial activity against resistant strains of bacteria such as MRSA, methicillin-resistant *S. epidermidis* (MRSE), vancomycin-resistant *Enterococcus faecium* (VREF), extended-spectrum beta-lactamase (ESBL) producing *P. aeruginosa*, and *Burkholderia cepacia*. The growth inhibition of MRSA and ESBL *E. coli* was found to have taken place at 6 hours of incubation with the Revamil^®^ honey. The RS honey demonstrated high sugar concentration, H_2_O_2_ production, MGO, low pH, and bee defensin-1, and these were significant factors contributing toward the bactericidal activity [12, 29].

Natural products include several phytochemical contents such as phenolic acid and flavonoids. These have been reported to have antioxidant and antibacterial activities. Moreover, the polyphenol compounds are essential for improving the potential effects on human health. Phenolic compounds are some of the most important groups that can be found in plants and honey. In addition, the composition of phenolic and flavonoid compounds of honey depends on floral sources, seasonal factors, and environmental factors [30]. As far as the quantitation of the total phenolic compounds in Thai honey is concerned, the phenolic compound was investigated using the Folin–Ciocalteu assay, and gallic acid was used as the standard. The assay measured the phenol and polyphenol derivatives, and other electron-donating antioxidants [31]. The results revealed that the total phenolic compounds existed in the wide range of 234.66 mg gallic acid/kg honey to 831.88 mg gallic acid/kg honey. These differences in the antioxidant activities of honey depend on the floral sources and the sources of collection. Furthermore, the total phenolic content of five different types of Yemeni honey demonstrated different values of antioxidant activity and phenolic compound, which can be attributed to the fact that the differences in the botanical sources of the honey and the colors of the honey were mainly due to the differences in the content and composition of the phenolic compounds in them [16, 32]. Monofloral Cuban honey showed phenolic content values ranging from 213.9 GAE/kg of honey to 595.8 GAE/kg of honey [33].

In this study, it was found that the honey from coffee flower had high phenolic content (734.76 mg gallic acid/kg honey) when compared to honey from other sources. The phenolic content of the honey from coffee flower was found to be as high as that of mānuka honey (831.88 mg gallic acid/kg honey). Thus, it can be concluded that different varieties of honey contain several different polyphenols, at different concentrations, which, in turn, affects the efficacy of the honey [34].

Flavonoids are widely found in food products derived from plant sources and honey, and they are a type of phenolic compounds [35]. The potential of flavonoids is considerable in its ability to protect the pBR322 plasmid DNA, against reactive oxygen species (ROS) or against H_2_O_2_-induced oxidative damage [36]. The investigation conducted in this study regarding the total flavonoid content of Thai honey showed that the flavonoid content depended on the floral sources as well as on the sources of collection. The honey collected from coffee flower had the highest flavonoid content, which was not significantly different from that of mānuka honey. Therefore, in this study, the observation regarding total flavonoid content correlated with that of total phenolic content. These results are similar to the findings obtained in the study conducted by Alvarez-Suarez *et al.* (2010) [33].

In addition, the correlation between total flavonoid content and total phenolic content depends on the color of fresh honey, for example, darker-colored varieties of honey (buckwheat and heather) showed total phenolic contents higher (71.7 μg/g to 202.6 μg/g) than those of lighter-colored honey (rape honey). However, after long periods of storage the color of the honey was observed to become too dark from hydroxymethylfurfural (HMF) [32]. Thus, antioxidant activity is also influenced by the temperature of processing and the storage method of the material. Documents on the varieties of African honey and the varieties of European honey certify them as containing flavonoids such as quercetin, hesperetin, kaempferol, apigenin, isorhamnetin, and myricetin [37].

The antioxidant activities of honey from different types of pollen were evaluated using DPPH and FRAP assays. Both the assays showed that honey from coffee flower had the highest antioxidant capacity compared to other types of honey. Similar antioxidant properties were also reported from Turkish red pine honey produced by *Marchalina hellenica*, Saudi Arabian honey, Peruvian honey, Malaysian tualang honey, American buckwheat honey, Spanish honey, Portuguese honey, Cuban honey, Venezuelan honey, and Ecuadorian honey [38–42]. Additionally, the antioxidant properties of the types of honey produced by stingless bees, for example, *Trigona carbonaria* in Australia and *Trigona laeviceps* in Thailand, were also reported [43, 44]. The antioxidant activity is due to the presence of various substances such as enzymes, organic acids, amino acids, Maillard reaction products, phenolic compounds, flavonoids, tocopherols, catechins, ascorbic acid, and carotenoids [45]. The high antioxidant capacity of honey correlated with the presence of phenolic and flavonoid compounds. Schneider *et al.* (2013) [19] also found a strong correlation between antioxidant capacity and polyphenol content since polyphenols are the major contributors to the antioxidant effect of honey.

Anti-tyrosinase activity of honey was tested and it showed that the percentage of inhibition ranged from 22.71 ± 6.82 - 87.73 ± 5.33%. Other natural substances such as ginger species and longan fruit extracts also showed anti-tyrosinase activity. Previous study demonstrated that favonols, galangin, kaempferol and quercetin were found to inhibit tyrosinase enzyme [46–48]. Thus, honey demonstrated efficacy to inhibit tyrosinase enzyme.

From the result in this study, the honey from coffee pollen showed high degree of phenolic, flavonoid, antioxidant and anti-tyrosinase activity, which was similar to mānuka honey. Therefore, the antioxidant and anti-tyrosinase activity of Thai and mānuka honey were correlated. Although, in this study the presence of phenolic and flavonoid compounds were varied depending on the types of honey but similar anti-bacterial activity was also observed on different types of honey.

Moreover, treatment of all Thai honey used in this study including honey from pollens of lychee, longan, sesame, polyflora, sunflower, forest flower, and coffee with catalase enzyme showed the removal of the antibacterial activity in this study. Therefore, antibacterial activity of Thai honey was due to hydrogen peroxide activity of the honey. The highest antioxidant activity of honey from coffee pollen was from phenolic and flavonoid compounds since they were the most important groups of the antioxidant substances in honey [16]. The antibacterial activity was not correlated with phenolic and flavonoid compounds that found in honey.

However, honey from coffee pollen did not show high anti-bacterial activity as the mānuka honey. The antibacterial compound of Thai honey was different from mānuka honey since the antibacterial activity of mānuka honey was mainly from non-peroxide component such as methylglyoxal [10].

The removal of hydrogen peroxide activity from Ulmo honey from Chile was also shown to reduce its antimicrobial activity when tested in the presence of catalase [49]. Therefore, Thai honey and mānuka honey showed different degree of anti-bacterial activity against pathogenic bacteria causing skin diseases.

## Conclusion

This study demonstrated the similar antibacterial activity of Thai honey obtained from various types of flowers against pathogenic bacteria causing skin diseases, including *S. aureus*, MRSA, *Corynebacterium* sp., and *P. acnes*. Antibacterial activity of Thai honey was due to peroxide activity, whereas the activity of mānuka honey was from non-peroxide activity of the honey. In addition, it was demonstrated that the honey collected from coffee flower showed the highest antioxidant activity and anti-tyrosinase activity, a finding that correlates with the phytochemical composition of honey which consists of phenolic and flavoniod compounds. Therefore, as is evident, in this study, it was also established that the potential of honey for use as a powerful source of antibacterial, antioxidant and anti-tyrosinase activity is tremendous. Moreover, honey can be used as an alternative therapeutic agent against pathogenic bacteria causing skin diseases.

## Methods

### Honey collection and preparation

The honey was collected from different sources and types of pollens, including lychee, longan, sesame, polyflora, sunflower, forest flower, and coffee during February - April 2012. Thai honey samples were purchased from local market and bee farm in July 2012. Also, mānuka honey (comvita®), produced in New Zealand, was used as the positive control in this study. Honey sample was kept in the dark at room temperature. The control solution contained 39% (w/v) d-fructose, 31% (w/v) d-glucose, 8% (w/v) maltose, 3% (w/v) sucrose, and 19% (w/v) water, and it was kept in refrigerator [28].

### Bacterial strains

Pathogenic bacteria causing infectious diseases on skin were tested. Gram positive bacteria, such as *Staphylococcus aureus*, methicillin resistant *S. aureus* (MRSA), *Staphylococcus epidermidis*, *Corynebacterium* sp., *Bacillus* sp., *Micrococcus luteus,* and *Propionibacterium acnes*, and Gram negative bacteria, such as *Pseudomonas aeruginosa,* were obtained from the Microbiology Section, Department of Medical Technology, Faculty of Associated Medical Sciences, Chiang Mai University, Chiang Mai, Thailand, while *P. acnes* DMST14916 were obtained from the culture collection section of the Department of Medical Science, Ministry of Public Health, Thailand. All the bacteria were cultured in a liquid medium for 24–72 hours before testing with honey.

### Viscosity and pH of honey

Viscosity was measured using a rotational viscometer (Brookfield viscometer DV-III+ Rheometer, USA) with SC4-29 spindle. The pH of honey was also measured by pH meter (Denver instrument, USA).

### Determination of activity of honey on bacteria using the agar well diffusion assay

Screening of the antibacterial activity of honey was performed using the agar well diffusion assay. The cultured bacteria were adjusted to McFarland No. 0.5 (1 × 10^8^ CFU/ml) with 0.85% NaCl solution. Then, the Mueller Hinton agar (MHA) plate was swabbed with the suspension. After that, 60 μl of the 100% honey sample and the control sugar solution were added to each well, by comparing it to phenol, which was used as the positive control. Gentamycin was also used as a drug control. The peroxide activity of honey on bacteria was also determined by the agar well diffusion assay after treatment of the 100% honey (100 μl) with 5 μl of catalase enzyme, 2 mg/ml (Sigma, 4000 U/mg) [50]. The plates were incubated at 37°C for 18–24 hours. After incubation, the inhibition zones were determined and the diameters of the zones were recorded.

### Determination of minimum inhibitory concentration (MIC) and minimum bactericidal concentration (MBC) of honey on bacteria

The minimum inhibitory concentration (MIC) was determined using the broth dilution method. The different samples of honey were diluted two-fold, from 100% to 50%, 25%, 12.5%, 6.25%, and 3.12%, with sterile Mueller Hinton broth (MHB). After that, the bacterial culture was adjusted to McFarland No. 0.5 for approximately 1 ×10^8^ CFU/ml. Thereafter, the bacterial culture was added to each dilution of honey and incubated at 37°C for 18–24 hours. After the incubation, the turbidity of the solution was observed and recorded as the lowest concentration of honey to inhibit the bacterial growth. The culture that completely inhibited visible growth on account of honey was streaked on an MHA plate for evaluating the minimum bactericidal concentration (MBC) after incubation at 37°C 18–24 hours. The MBC was determined as the minimum bactericidal concentration.

### Determination of time–kill endpointsof honey on bacterial growth

The time–kill studies were performed for a period of 24 hours, and stock honey was prepared to 2 MIC. Then, the bacteria were adjusted to McFarland No. 0.5 and mixed with honey in the ratio of 1:1 and incubated at 37°C, while inoculums without honey were used as the control. The samples were taken from the experiment at 0 minutes, 10 minutes, 20 minutes, 30 minutes, 90 minutes, and 120 minutes, and every 2 hours until 24 hours. At the end of each time period, the sample was ten-fold serial diluted with 0.85% NaCl, and 100 μl of each dilution was spread onto the MHA plates. After incubation at 37°C for 24 hours, the bacterial colonies were counted and recorded.

### Determination of total phenolic content of honey

The Folin–Ciocalteu method was used to determine the total phenolic content [35]. Honey (250 μl) at a concentration of 100 mg/ml was mixed with 1.25 ml of water, 250 μl of 95% ethanol, and, then, 125 μl of 50% Folin–Ciocalteu. The solution was incubated at room temperature for 5 minutes. After that, 5% of Na_2_CO_3_ was added to the solution and incubated for 1 h, and the reaction was measured to determine the absorbance at the wavelength 725 nm. The total phenolic content was expressed in mg of gallic acid equivalents (mg GAE/kg of honey).

### Determination of flavonoid content of honey

The total flavonoid content was determined using the colorimetric method discussed in Ghasemi *et al.* (2009) [35]. The honey sample was diluted with methanol to a final concentration of 100 mg/ml. Five hundred microliters of the honey solution was mixed with 0.1 ml of 10% aluminium chloride, 1.5 ml methanol, 0.1 ml 1 M potassium acetate, and 2.8 ml distilled water. Thereafter, the solution was incubated at room temperature for 30 minutes. After the incubation, the absorbance of the reaction was measured at the wavelength 415 nm. The total flavonoid content was expressed as mg quercetin equivalents (mg quercetin/kg of honey).

### Determination of antioxidant activity of honey by 2, 2-diphenyl-1-picrylhydrazyl (DPPH) assay

The antioxidant activity of the different types of honey was evaluated using the DPPH radical scavenging assay [35]. The honey was prepared to various concentrations by dissolving in methanol. A volume of 0.5 ml of the honey was mixed with 1.5 ml of the DPPH reagent and incubated in the dark at room temperature for 20 minutes. The absorbance of the reaction was measured at the wavelength 517 nm. The DPPH without honey was used as the control and methanol was used as the blank solution. The scavenging activity was evaluated. The IC_50_ (mg/ml) value was calculated using the following equation:


Finally, the antioxidant activity of honey was reported as the gallic acid equivalent antioxidant capacity (mg GAE/kg of honey).

### Determination of antioxidant activity of honey by ferric reducing antioxidant power assay (FRAP)

The FRAP assay was performed according to the method discussed in Benzie and Strain (1996) [51]. The different samples of honey were dissolved with deionized water to 100 mg/ml. The FRAP reagent included 30 mM acetate buffer pH 3.6, 10 mM TPTZ solution, 20 mM FeCl_3_ solution, and deionized water. A volume of 0.5 ml of the honey solution was mixed with 1.5 ml of the FRAP reagent. Thereafter, the mixture was incubated in the dark at room temperature for 15 minutes. The absorbance of the reaction was measured at the wavelength of 593 nm. The FRAP without honey was used as the control and deionized water was used as the blank solution. The antioxidant activity of honey was calculated from the ferrous sulfate standard curve, which has the antioxidant standard, and reported as mg ferrous sulfate/kg of honey.

### Determination of anti-tyrosinase activity of honey

The method was evaluated using the dopachrome micro-plate [52]. Honey was dissolved in 20% ethanol to a final concentration of 50%. This stock solution, 50 μl was combined with 150 μl of 0.02 M phosphate buffer (pH 6.8) and 50 μl of mushroom tyrosinase (313 Units/ml in phosphate buffer, Sigma Chemical), and incubated for 10 minutes. After that, 0.32 mM 3,4-Dihydroxy-L-phehylalanine (Sigma Chemical), 50 μl was used as substrate and added to the each well. Anti-tyrosinaseactivity was evaluated by measuring absorbance at 492 nm before incubation at 25°C and after incubation for 2 min. Kojic acid (Merck Millipore) was used as the standard inhibitor of tyrosinase enzyme. The % inhibition was calculated using the following equation:


Where A was the optical density (OD_492_) of the control (L-Dopa mixed with tyrosinase enzyme in buffer); B represented the blank (L-Dopa in buffer); C represented the reaction ofL-Dopa with tyrosinase enzyme and honey in buffer and D represented the blank of C (L-Dopa mixed with honey in buffer)

### Statistical analysis

The results were expressed as mean ± standard deviation. The ANOVA test was used to analyze the variance of data (SPSS software version 17.0 for Windows). The analysis average of the treatment using multiple comparisons was determined by using Dancan’s multiple range tests, and the data were compared using the p values: p < 0.05 was considered statistically significant. The least significant difference (LSD) was used to determine the difference between the methods used to the investigation of the various antioxidant capacities.
